# Stool Microbiome Features and Weight Change Response to Treatment for cancer cachexia

**DOI:** 10.1002/jcsm.13816

**Published:** 2025-05-05

**Authors:** Rima Nasrah, Mary Kanbalian, Christina Van Der Borch, Ken Dewar, Stéphanie Chevalier, R. Thomas Jagoe

**Affiliations:** ^1^ McGill Cancer Nutrition Rehabilitation Program Jewish General Hospital (CNR‐JGH) Montreal Canada; ^2^ Peter Brojde Lung Cancer Centre Segal Cancer Centre, Jewish General Hospital Montreal Canada; ^3^ Division of Experimental Medicine, Faculty of Medicine and Health Sciences McGill University Montreal QC Canada; ^4^ McGill Centre for Microbiome Research McGill University Montreal QC Canada; ^5^ Department of Human Genetics McGill University Montreal QC Canada; ^6^ Research Institute of the McGill University Health Centre Montréal QC Canada; ^7^ School of Human Nutrition McGill University Ste‐Anne‐de‐Bellevue QC Canada; ^8^ Department of Medicine McGill University Montreal QC Canada

**Keywords:** cancer cachexia, gut microbiome, multidisciplinary cachexia clinic, nutritional counselling, predictive biomarkers, weight change

## Abstract

**Background and Aims:**

Cancer cachexia is characterised by significant weight loss and muscle wasting that adversely affects patient outcomes. Nutritional interventions in cancer cachexia leads to improved outcomes, including improved weight change. However, there are wide variations in weight response to dietary interventions. Thus, it remains difficult to predict response to a given increase in dietary intake at an individual patient level. This study aimed to identify gut microbiome features that could serve as potential predictive biomarkers for response to individualized dietary intervention in patients with cancer cachexia attending the McGill Cancer Nutrition‐Rehabilitation Program at the Jewish General Hospital (CNR‐JGH).

**Methods:**

Participants were recruited from CNR‐JGH clinic. Interventions included individualized nutritional counselling by a registered dietitian, to increase energy and protein intake to meet recommended levels. Stool DNA samples were collected at baseline (V1) and visit 2 (V2), and gut microbiome profiles were analysed to assess microbial diversity and identify differentially abundant genera in patients who lost weight (WL, *N* = 8) vs. maintained/gained weight (WSG, *N* = 29) at subsequent CNR‐JGH clinic visits.

**Results:**

Greater alpha‐diversity and higher *Lachnospira* genus abundance at baseline predicted higher likelihood that patients would have good response to CNR‐JGH intervention (WSG at V2). Though predictors of poor response to nutritional intervention (WL at V2) were not identified, subjects in the WL group exhibited lower alpha‐diversity and greater microbial population instability after CNR‐JGH interventions.

**Conclusions:**

In this cohort of patients with cancer‐related weight loss attending a cancer cachexia clinic, certain gut microbiome features were associated with response to dietary interventions. Patients who lost weight after CNR‐JGH intervention also developed a less diverse and less stable gut microbiome. *Lachnospira* genus abundance is a potential predictor of positive weight change response to dietary intervention as part of multimodal care for cancer cachexia, and further confirmatory studies are warranted. In addition, targeted dietary approaches to maintain diversity and gut microbiome population stability may have a role in improving the response to dietary interventions in cancer cachexia.

**Abbreviations**
WL: weight loss
WSG: weight stable/gain
CNR‐JGH: McGill Cancer Nutrition‐Rehabilitation Program at the Jewish General Hospital
V1: visit 1
V2: visit 2
GM: gut microbiome
CRP: C‐reactive protein
BMI: body mass index
ALM: appendicular lean mass
DXA: dual energy x‐ray absorptiometry
HEI‐2015: Healthy Eating Index – 2015 version
PCR: polymerase chain reaction
QIIME2: Quantitative Insights into Microbial Ecology 2
ASV: amplicon sequence variant
16S rRNA sequencing: 16S ribosomal RNA sequencing

## 1 Introduction

Weight loss and muscle wasting are common in patients with advanced cancer and are key features of cancer cachexia. The presence of cancer cachexia is associated with higher rates of intolerance or complications of cancer treatment and increased mortality. Despite the high prevalence and clinical importance of cancer cachexia, there is no broadly accepted standard of care for affected patients and no approved medication that can reliably reverse this condition. Instead, there is growing consensus that management of cancer cachexia requires a multidimensional approach, including optimizing dietary intake [1, 2].

A recent large multi‐centre cohort study showed that reduced food intake was the most important single determinant of cancer‐related weight loss [3]. Data from the CNR‐JGH, which specializes in the management of patients with cachexia, showed that 27% of patients with cachexia are consuming diets that would likely be insufficient to maintain weight even in healthy individuals [4]. Current dietary guidelines in oncology have proposed higher targets for energy and protein intake to accommodate the increases in basal metabolic rate and protein losses exhibited by some patients. However, on admission to the CNR‐JGH clinic only 18% of patients were meeting these recommended levels of intake [4]. Multidisciplinary interventions including individualized nutritional counselling in the CNR‐JGH clinic, increased nutritional intake and correlated with stabilisation of weight. Furthermore, there was a highly significant positive correlation between dietary energy or protein intake and weight change, both before and after patients were being seen within the clinic [4]. These data suggest that an active nutritional intervention is an important factor in preventing and even reversing cancer cachexia.

Several systematic reviews have summarized the evidence supporting the use of nutritional interventions to combat cancer‐related weight loss in different patient populations [5, 6, 7, 8, 9]. Overall, nutritional interventions lead to improved nutritional intake, body weight, body composition, nutritional status scores and quality of life. However, the overall clinical effect is often small [8]. Furthermore, the benefits for any specific outcome of interest (e.g., body weight, lean mass) were not observed in all studies. Methodological differences and certain weaknesses in study design may explain some of the variation in results, including lack of individualized supplementation plans, poor subject compliance or failure to achieve positive energy balance despite nutritional supplementation [10, 11, 12]. However, it is now clear that there are other unidentified patient‐specific factors which modulate the response to nutritional counselling and dietary intervention [13]. Thus, it remains very difficult to predict an individual patient's response to a given increase in dietary intake [4]. Indeed, despite the plethora of methods to identify and categorize patients with nutritional depletion, there are still no validated tools to predict response to nutritional interventions in any field of clinical nutrition, including oncology.

The inability to accurately predict response to dietary interventions in cancer cachexia is a major barrier to effective management. Accurate, early identification of individuals needing more aggressive approaches to overcome anabolic resistance to nutritional interventions would be immensely useful in treatment planning. Thus, new approaches are needed to characterise patients' ability to respond to nutritional interventions. In addition, closer study of the factors distinguishing patients with different nutritional responses may also point the way towards new therapeutics to facilitate weight gain with nutritional interventions in cancer cachexia.

The gut microbiome (GM) is emerging as a bioactive system that plays a major role in nutrient and energy metabolism, appetite regulation, and inflammation, all of which are relevant to the development and progression of cancer cachexia [14]. In obesity, a study of weight loss in response targeted dietary interventions in overweight middle‐aged adults showed that predictive models including specific gut microbial genera, urine metabolites and type of diet performed better than a diet‐only model [15]. Similarly, GM composition explained around 25% of the variation in weight loss response after an 8‐week low‐energy diet in pre‐diabetic adults [16]. In oncology, patients with lung cancer and cachexia were found to have GM profiles that distinguished them from non‐cachectic lung cancer patients [17]. Furthermore, in pancreatic cancer patients receiving 12 weeks of enteral feeding, the increased abundance of the *Veillonella* genus in stool at baseline was associated with subsequent weight stability rather than weight loss [18]. Thus, the GM may be an important determinant of nutritional response to dietary interventions in cancer. However, to date, studies focused on GM predictors of response to diet‐based nutritional counselling in patients with cancer cachexia have not been performed.

The current study was designed to determine the GM composition of patients with cancer‐related weight loss before and after nutritional interventions delivered within an expert multidisciplinary cancer cachexia treatment team. The goal was to identify features of the GM which identified patients who subsequently lost weight despite individualized nutritional interventions.

## 2 Methods

### 2.1 Study Design and Participant Recruitment

The study was performed in the Cancer Nutrition Rehabilitation clinic at the Jewish General Hospital (CNR‐JGH) in Montreal. This is a clinic specializing in the management of cancer cachexia, based in a McGill University Teaching Hospital [4]. Briefly, patients with weight loss, anorexia, or generalised functional decline are seen by a team comprised of a physician, nurse, physiotherapist and a dietitian. At each visit, patients are evaluated by each professional, and an inter‐disciplinary intervention plan is developed to address any barrier symptoms (e.g. anorexia, nausea) and to optimise dietary intake and functional status using individualized nutritional counselling and exercise training programs. Typically, patients attend CNR‐JGH clinics at 6‐week intervals, but this schedule is adjusted depending on clinical need and patients' availability. All patients receive individualized nutritional counselling by a CNR‐JGH dietitian at each visit to achieve target intakes of at least 30 kcal/kg body weight and 1.3 g protein/kg body weight. Primary dietary recommendations typically include use of energy and protein‐dense foods and beverages, increasing meal frequency with or without the use of liquid nutritional supplements. Nutritional advice is adapted to individual's usual diet, personal eating patterns, preferred food consistency and medical conditions, as well as specific symptoms (e.g., dysphagia, constipation, diarrhoea, altered taste and temperature sensitivity).

Study participants were recruited from patients referred to the CNR‐JGH clinic. In addition to standard nutritional and clinical testing, participants were asked to provide stool samples for gut microbiome (GM) analysis at entry to the study (V1) and after approximately 6 weeks of CNR‐JGH intervention (V2). Inclusion criteria were adults of ≥ 18 years with a cancer diagnosis capable of writing/reading in English or French attending CNR‐JGH clinic. Exclusion criteria were: a) having a colostomy or ileostomy, b) clinical evidence of malabsorption (e.g., untreated exocrine pancreatic enzyme deficiency), c) current or recent use of antibiotics, d) cognitive barriers (i.e., dementia) and e) having ascites/edema masking real weight change. All eligible patients were invited to participate, and all participants were given a detailed explanation of the study and signed an informed consent form. This study was approved by the Research Ethics committee of the Jewish General Hospital (Approval# 15‐098).

### 2.2 Analysis Plan and Definitions of Clinical Groups

The primary outcome was to compare the gut microbiome (GM) composition from stool samples collected at baseline (V1) in participants who lost weight versus those who remained stable or increased weight at their second CNR‐JGH visit (V2). Weight change categories were defined as: a) weight loss ≥ 1 kg (WL), b) weight stable, ± < 1 kg (WS), and weight gain ≥ 1 kg (WG). For most analyses, the WS and WG groups were combined (WSG) and compared to those who lost weight (WL).

A secondary outcome was the comparison of GM composition at V2 in WSG vs. WL participants, to determine if differences in GM became evident after dietary intervention in the two groups. In addition, GM composition at V1 and V2 was compared in participants who went on to gain/maintain vs. lose lean mass during the CNR‐JGH study intervention.

Exploratory outcomes included the relationship between GM and other potentially important clinical factors related to cancer cachexia including combined diet‐weight change groups (a. participants eating an adequate diet but losing weight, b. those eating an adequate diet and gaining weight as expected and c. those who are eating a poor diet), prior weight change, energy and protein intake, dietary quality, markers of systemic inflammation, key barrier symptoms such as anorexia, body composition changes including appendicular lean mass, (ALM) and use of certain medications for comorbidities including diabetes.

Two different approaches were used to analyse the microbiome and compare groups of interest. These analyses were designed to capture global microbiome features, including diversity and population stability at each time point as well as to identify differential occurrence of specific sequences or taxa. In each case, both standard taxonomy‐based analysis and analysis using sequence counts was used and are described in detail below.

### 2.3 Collection of Clinical Data

Comprehensive clinical and nutritional data was collected as part of usual CNR‐JGH routine clinical evaluation as detailed elsewhere [4]. Patient‐reported weight change history prior to V1 was collected and corroborated using medical records where available. Weight and height were measured at each visit using the same scales after removal of shoes and coats or jackets and emptying pockets. Dietary intake was determined by the CNR‐JGH dietitian using the patient's report of intake over the prior 24‐h period (24‐h recall) or, in selected cases where this approach was difficult, an estimate of current usual intake in 24‐h was determined by interview. Diet recalls were analysed using a commercial software (the Food Processor, ESHA, 2009) with the Canadian Nutrient File. Energy intake was categorized as follows: a) low‐energy intake, which is likely not meeting estimated energy needs for cancer patients (<25 kcal/kg); b) moderate intake, which is likely meeting current guidelines [2] in patients with cancer to maintain weight (25‐30 kcal/kg); and c) a high intake, which is likely sufficient for weight gain ( > 30 kcal/kg). Protein intake was categorized in a similar fashion (high intake > 1.3 g/kg, low is < 1 g/kg and moderate is in between high and low). In addition, dietary data were evaluated for dietary quality before and after CNR‐JGH intervention using the National Institute of Health (NIH) Automated Self‐Administered 24 h recall (ASA24‐Canada version 2018) to calculate Healthy Eating Index (HEI‐2015). Details of cancer treatment type were updated at each visit. All other medications were also recorded for each patient including metformin, proton pump inhibitors, statins, laxatives, loperamide anti‐diarrheal drug and antibiotics, all of which have been proposed to have some potential impact on the gut microbiome [19, 20].

### 2.4 Body Composition Measurements

Dual energy x‐ray absorptiometry (DXA) was used to measure total body composition and regional fat and lean soft tissue mass, including ALM. Body composition measurements were expressed relative to NHANES reference values [21] specific for sex, age and ethnicity. Participants were also categorized as having sarcopenia based on previously used sex‐specific definitions in the Canadian population [22]. See Data S1 for details.

### 2.5 Faecal Samples, DNA Extraction and 16S rRNA Amplicon Sequencing

#### 2.5.1 Faecal Sample Collection

Stool samples for GM analysis were collected from each participant at V1 and V2 at least 6 weeks apart. Samples were collected using OMNIgene GUT stool kits (DNA Genotek Inc. Ottawa, Ontario, Canada). Participants were instructed to collect their stool samples at home within 1 week of clinic visit. Most stool samples were returned in person to the study coordinators at CNR‐JGH and a minority were mailed or collected from the participants' homes. Additional quality assurance standards were applied to ensure stool samples were contemporary with the clinical evaluations: samples received > 2 weeks after the clinic visit were excluded. Technical controls to check the quality of sequence data for each run were also used as described below.

#### 2.5.2 DNA Extraction and 16S rRNA Gene Next‐Generation Sequencing

DNA extraction from stool samples was performed at the microbiology department at the Jewish General Hospital. DNA extraction was performed using EazyMag (Biomerieux, Saint‐Laurent, Quebec, Canada), following the stool isolation protocol as previously described [23] and immediately stored at −80°C. The concentration of isolated nucleic acid in each aliquot was quantified (Nanodrop, ThermoFisher Scientific) and 20 uL of each aliquot was used for sequencing. PCR amplification of the fourth hypervariable region (V4) of the bacterial 16S ribosomal RNA gene was performed at the McGill University and Genome Quebec Innovation Centre using the 515/806R primer set. 16S rRNA sequencing was performed on Illumina MiSeq PE250 using golay barcodes, producing 250 bp paired‐end reads.

### 2.6 Bioinformatics Analysis

#### 2.6.1 16S rRNA Gene Sequence Data Processing

Detailed methods of the sequence analyses are included in the Data S1. Briefly, raw sequences were processed using the Quantitative Insights into Microbial Ecology 2 (QIIME2) pipeline (version 2022.8 and 2024.5). Sequences were demultiplexed, quality‐filtered using q2‐demux followed by DADA2 plug‐ins to obtain the final amplicon sequence variant (ASV) table. The FastTree method in QIIME using the ‘maaft’ method was used to create a rooted phylogenetic tree as required for calculation of two diversity metrics (Faith Phylogenetic Diversity alpha diversity index, and Weighted/Unweighted UniFrac beta‐diversity). A sampling depth of ~18 000 reads per sample was chosen for the diversity analysis. Relative abundances of taxa were examined using a pre‐trained Naïve Bayes classifier and the q2‐feature‐classifier plugin on QIIME2, which was trained on the Greengenes 13_8 99% OTUs.

#### 2.6.2 Gut Microbiome Diversity Analyses

Alpha diversity analyses were measured to compare GM diversity of different clinical groups (i.e., WSG vs. WL) at each clinic visit (V1 and V2) using four diversity statistics in QIIME2: Shannon (Observed OTU's), Faith's phylogenetic diversity **(**Faith PD), and Pielou's Evenness index. After calculating alpha‐diversity for each of the individual's GM sample, comparison between groups of interest was performed. Beta‐diversity (B‐diversity) was measured using four indices in QIIME2: Jaccard distance, Bray‐Curtis, Unweighted Unifrac, and Weighted Unifrac indices. These indices are direct measures of (dis)similarity between two GM samples and were used to look at potential differences in the change in GM for each paired sample (V1‐V2) for participants in WSG vs. WL. See Data S1 for details.

#### 2.6.3 Global Changes in Detected ASV

The processed ASV data were analysed as follows: A) Analysis of the most abundant ASV: The number of unique ASVs making up 80% of the total cumulative counts was determined for each participant sample. The number of these ‘most abundant’ ASVs was compared between weight change groups. B) Analysis of the stability of ASV counts between V1 and V2. The number of ASVs where large changes in abundance (count change > 500) were determined for all paired participant samples (V1‐V2). The number of these ‘top change’ ASVs was compared between weight change groups.

#### 2.6.4 Differential Abundance Analysis

Differential abundance testing was performed with analysis of composition of microbiomes with bias correction (ANCOM‐bc) [24] QIIME2 plug‐in to identify differences in the bacterial composition of the microbiomes sample between different clinical groups. The Holm‐Bonferroni method was used to adjust for multiple hypothesis testing, and adjusted *p*‐value < 0.05 were considered significant. The analysis was performed using both relative frequency of ASV and detected genera as input. To reduce noise, a prevalence filter to remove ASVs detected in less than 10% of samples was applied [25].

#### 2.6.5 Sequence Data Quality Control

In addition to the study samples obtained from participants, four different types of controls were sequenced in parallel with the faecal samples from participants. These include testing negative controls (to test background contamination of false positive reads), within‐run reproducibility of PCR sequencing using DNA extract aliquots of the same subject, and tracking drift between sequencing runs by comparing taxonomy results of same samples ran by different sequencers.

Finally, DNA extraction reproducibility was tested by comparing different aliquots of the same stool sample extracted on different days. In brief, one stool sample from a healthy volunteer was homogenized, aliquoted and stored at −80C. DNA extraction for one aliquot was then performed every time an incoming stool sample from a participant was processed. In total, 64 of these DNA extraction control aliquots were processed. Alpha diversity of ASVs obtained for all control aliquots was similar, but the assigned taxonomy at the genus level was clearly qualitatively different for two samples. The stool samples which were sequenced on these two runs were excluded from further analysis. See Data S1 for details.

### 2.7 Statistical Analysis

Spearman correlation analysis was used for continuous variables and gut microbiome analysis (i.e., alpha diversity). Kruskal‐Wallis analysis of variance was used for comparing categorical metadata, and when significant, pairwise comparison was carried out by Mann–Whitney U test. Wilcoxon signed‐rank test was used when comparing individuals within the same group. To correct for multiple comparison testing, the Benjamini‐Hochberg correction was applied for diversity analysis and the Holm‐Bonferroni was applied for differential abundance testing (ANCOM‐bc), and FDR‐adjusted *p*‐value < 0.05 was considered statistically significant. Between‐group differences were assessed using Fisher's exact tests for categorical data. Conditional probability analysis was used to identify the ability of specific baseline factors or conditions to predict whether subjects were classified as WSG vs. WL after CNR‐JGH intervention (https://en.wikipedia.org/wiki/Conditional_probability). Statistical analyses were performed with QIIME2 and R using R Studio (version 2022.07.01) and plots were prepared using ggplot2. In addition to significance testing for each diversity metric, a concordance threshold was set requiring a minimum of two out of the four diversity metrics to be statistically significant for an overall difference in diversity to be declared.

## 3 Results

### 3.1 Recruitment to Study

**FIGURE 1:**
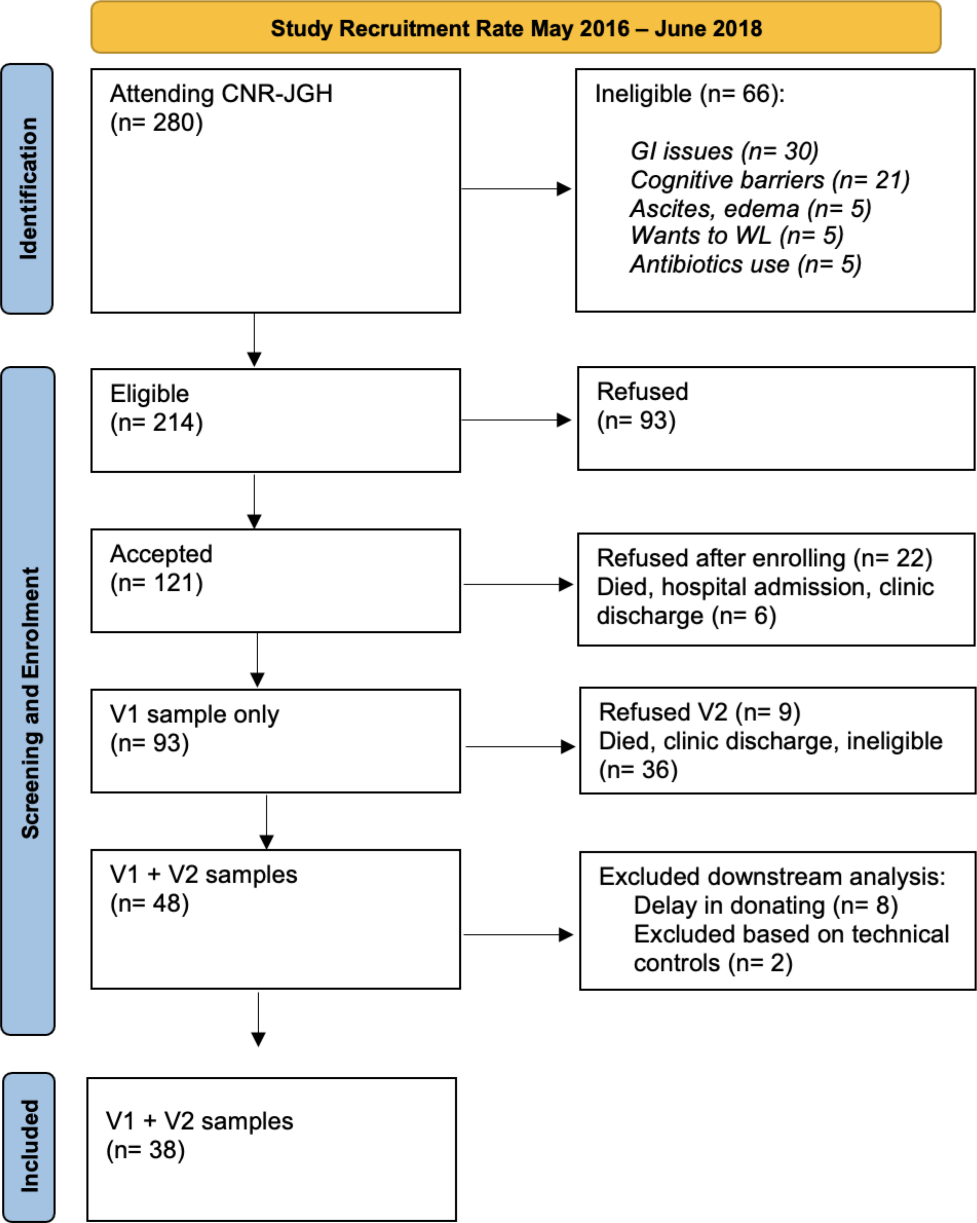
Participant recruitment (May 2016–June 2018). PRISMA flow diagram of patient screening and participant recruitment. See Methods for explanation of categories for subjects excluded from the study.

Subject recruitment took place from May 2016 to June 2018 (Figure 1). Of 280 patients screened for the study, 121 consented, and after exclusion of ineligible stool samples, 38 paired participant samples were retained for data analysis (Figure 1).

### 3.2 Stool Samples Retained for Inclusion in Analysis and Justification

Of the 93 participants who donated one stool sample, 45 did not provide a second sample. These included participants who withdrew from the study after V1 (*N* = 9). Other reasons included death (*N* = 7), no further follow up in CNR‐JGH clinic due to either debility or poor motivation (*N* = 16), clinical improvement (*N* = 10) or unknown reason (*N* = 2), and withdrawal because participant became ineligible for the study (N = 1). Of 48 participants who donated paired stool samples, i.e., at V1 and V2, eight were excluded as one or more sample was not received within 2 weeks of clinic date. Two further participant samples were excluded as they failed the sequence quality control procedures (Figure 1).

### 3.3 Baseline Characteristics of Participants and Anthropometric Measures

Baseline characteristics of participants are found in Table 1. The participants fell into the following weight change groups for analysis of primary outcomes. Weight gain (WG) *N* = 19, weight stable (WS) *N* = 10 and weight loss (WL) *N* = 8. One additional valid participant paired sample set could not be categorized by weight change due to missing data as the subject refused to be weighed at one visit. Results of this sample were included in analyses of other outcomes where appropriate.

**TABLE 1:** Baseline characteristics of all participants, stratified by weight change category.

			All ^a^ (*N* = 38)		
				WSG (N = 29)	WL (N = 8)
Age (y)	*Mean (SD)*		66 (13)	65 (12)	70 (12)
Sex—male	*N (%)*		21 (55%)	16 (55%)	5 (63%)
Cancer type	*N (%)*	GI	8 (21%)	5 (17%)	3 (38%)
		Haematological	6 (16%)	4 (14%)	2 (25%)
		Lung	5 (13%)	5 (17%)	0
		Other^b^	19 (50%)	15 (52%)	3 (38%)
Cancer stage	*N (%)*	II	3 (8%)	2 (7%)	1 (13%)
		III	4 (11%)	4 (14%)	0
		IV	28 (74%)	20 (69%)	7 (88%)
Cancer treatment Line	*N (%)*	0	6 (16%)	4 (14%)	2 (25%)
		1	19 (50%)	16 (55%)	2 (25%)
		≥2	13 (34%)	9 (31%)	4 (50%)
ECOG	*N (%)*	0–1	34 (89%)	27 (93%)	6 (75%)
		2	4 (11%)	2 (7%)	2 (25%)
Cachexia code^(1)^	*N (%)*	None	5 (13%)	5 (18%)	0
		Pre‐cachexia	4 (11%)	3 (10%)	0
		Cachexia	27 (71%)	20 (69%)	7 (88%)
		NA	2 (5%)	1 (3%)	1 (12%)
WLGS ^(2)^	*N (%)*	0–2	10 (26%)	9 (31%)	0
		3	15 (39%)	11 (38%)	4 (50%)
		4	11 (29%)	8 (28%)	3 (38%)
		NA	2 (5%)	1 (3%)	1 (13%)
mGPS^(3)^	*N (%)*	0	22 (58%)	18 (62%)	3 (38%)
		1	9 (24%)	8 (28%)	1 (13%)
		2	5 (13%)	3 (10%)	2 (25%)
		NA	2 (5%)	0	2 (25%)

Abbreviations: WSG: Weight stable/gain, WL: Weight loss, WLGS: Weight Loss Grading System (see Methods for details), mGPS: modified Glasgow Prognostic Score, ECOG: Eastern Cooperative Oncology Group performance status.

^a^ One participant who could not be categorized by weight change due to missing data as the subject refused to be weighed at one visit. Results of this sample were included in analyses of other outcomes where appropriate.

^b^ Other cancer types: urogenital (*n* = 7), breast (*n* = 6), gynaecological (*n* = 2), mesothelioma (*n* = 1), other (*n* = 3).

The majority (74%) had metastatic cancer (stage IV) and had received either no treatment or first‐line cancer treatment only (66%). Most participants (89%) had well‐preserved performance (ECOG 0‐1) and fulfilled criteria for cachexia (71%) or pre‐cachexia (11%) [26]. The remainder were patients referred to CNR‐JGH with other features of anorexia–cachexia including fatigue and weakness, which are not encompassed by the current definition. There were no differences in baseline characteristics between the two main weight change groups of interest (WL vs. WSG).

The weight loss grading system (WLGS) [27] is a validated prognostic score for patients with cancer that combines weight loss and body mass index (BMI), where grade 0 comprises individuals with little on weight loss and high BMI and grade 4 are those with highest weight loss and lowest BMI. Using this system, 68% of participants were grade 3 or 4 (the two worst prognostic categories). Using a different prognostic scoring system based on presence of markers of systemic inflammation (the modified Glasgow Prognostic Score (mGPS)) [28], 24% had an intermediate score 1 (CRP levels > 10 mg/L) but only 13% had the worst prognostic score 2 (elevated CRP and hypoalbuminemia). Using the mGPS score, 10% of the WSG group, and 25% of the WL had the worst prognostic score (see Table 1). At baseline, BMI and recent weight loss history were similar for participants who were designated WSG vs. WL (Table 2). Approximately half of participants were sarcopenic at baseline (*N* = 26).

**TABLE 2:** Baseline BMI, body composition, and dietary intake of participants.

			All (N = 38)		
				WSG (N = 29)	WL (N = 8)
BMI (kg/m^**2**^)	*Mean (SD)*		24 (4)	24 (4)	23 (2)
Weight change (Δ kg) of UBW	*Mean (SD)*		−12.5 (10.2)	−12.7 (10.4)	−12.7 (11)
Weight change (Δ kg) 6 week pre‐V1 ^a^	*Mean (SD)*		−1.2 (3.4)	−0.8 (−2.8)	−2.7 (5.8)
Sarcopenia^b^ ^(4)^	*N (%)*	Sarcopenic	18 (69%)	15 (71%)	3 (60%)
		Non‐sarcopenic	8 (31%)	6 (29%)	2 (40%)
Energy intake (kcal/kg)	*Mean (SD)*		26 (10)	26 (10)	24 (7)
Protein intake (g/kg)	*Mean (SD)*		1.1 (0.4)	1.0 (0.4)	1.1 (0.2)
Energy intake categories^c^	*N (%)*	High	12 (32%)	9 (31%)	2 (25%)
		Moderate	7 (18%)	5 (17%)	2 (25%)
		Low	19 (50%)	15 (52%)	4 (50%)
Dietary quality score^d^	*N (%)*	V1	56 (14%)	55 (15%)	58 (10%)

*Note:* Welch's *t*‐test (unequal variance) between weight change subcategories (i.e., WSG vs. WL) was performed for: BMI, weight change, energy intake, protein intake, dietary quality scores; none of which was significant.

^a^ Weight change 6wk pre‐V1 is reported weight change over prior 6 weeks, other weight changes are measured.

^b^ Based on available body composition analyses *N* = 26.

^c^ Energy intake categories are defined as: high: consuming more than 30 kcal/kg, low: consuming less than 25 kcal/kg, and moderate is everything else. See Methods.

^d^ HEI‐2015 scores. For reference, age‐corrected HEI‐2015 mean scores for the U.S. population for a comparable group would be between 57 and 60. Dietary quality scores were based on *N* = 34 (*N* = 27 WSG, *N* = 7 WL).

Abbreviations: WSG: Weight stable/gain, WL: Weight loss, UBW: usual body weight, V1: Visit 1.

### 3.4 Changes in Weight, Body Composition, and Dietary Intake Between CNR‐JGH Visits (V1 to V2)

During the study period (V1 to V2), mean weight change for WL group was −2.9 (±1.3) kg compared with a 1.5 (±1.4) kg gain in WSG group (*p* < 0.001) (Table 3). Overall, the impact of nutritional counselling in CNR‐JGH clinic was confirmed by significant increases in both mean energy (26 ± 10to 30 ± 12 kcal/kg, *p* value = 0.003) and protein intake (1.1 ± 0.4 to 1.4 ± 0.6 g/kg, *p* value < 0.001) (Table 3). Half of participants were consuming a poor energy intake at V1 (52% and 50% in WSG and WL groups respectively). By V2, more participants had a high energy intake and only 35% and 13% of WSG and WL groups, respectively, were eating a poor ( < 25 kcal/day) diet. There was also no difference in average energy intake between WSG and WL. Similarly, the proportion of participants eating a higher protein intake ( > 1.3 g/kg) increased at V2 (from 31% to 59% for WSG; from 38% to 63% for WL).

**TABLE 3:** Changes in weight, body composition, and dietary intake during CNR‐JGH (V2‐V1).

			
				WSG (*N* = 29)	WL (*N* = 8)
Weight change (Δ kg)	*Mean (SD)*		0.6 (2.4)	1.5 (1.4)	−2.9 (1.3)***
ALM change ^a^ (Δ kg)	*Mean (SD)*		0.3 (±0.7)	0.4 (±0.7)	−0.5 (±0.6)
	*N (%)*	Gain	8 (47%)	8 (57%)	0
		Stable	5 (29%)	4 (29%)	1 (33%)
		Loss	4 (24%)	2 (14%)	2 (67%)
Energy intake V2 (kcal/kg)	*Mean (SD)*		30 (12)^$^	30 (13)^$^	29 (4)^$$$^
Energy intake changes (Δ kcal/kg)			8 (11)	6 (12)	12 (10)
Energy intake categories V2 ^b^	*N (%)*	High	16 (42%)	13 (45%)	3 (38%)
		Moderate	10 (26%)	6 (21%)	4 (50%)
		Low	11 (29%)	10 (35%)	1 (13%)
Dietary quality scores changes ^c^	*Mean (SD)*		3 (13)^d^	2 (17)	7 (18)

*Note:* See Methods for details on appendicular lean mass categories, total lean change categories, energy intake categories. Significance testing performed using Welch's *t*‐test: *** for *p* < 0.001 comparing means between WSG vs. WL Significance testing performed using Welch's *t*‐test: ^$^, ^$$$^ for *p* < 0.05, < 0.001 respectively comparing means between V1 and V2 within same clinical subgroup (i.e. V1 vs. V2 in WSG group).

^a^ Appendicular lean mass changes categorized as: high (gain of more than 0.5 kg between visits), stable (within 0.5 kg), and loss (loss of more than 0.5 kg). Based on available body composition records, *N* = 17.

^b^ Energy intake categories are defined as: high: consuming more than 30 kcal/kg, low: consuming less than 25 kcal/kg, and moderate is everything else. See Methods for details.

^c^ Change in HEI‐2015 scores.

^d^ HEI‐2015 scores were not statistically different between V2 and V1 for both weight change groups.

Abbreviations: WSG: Weight stable/gain, WL: Weight loss, ALM: appendicular lean mass, V2: Visit 2.

Dietary quality scores (as measured by HEI‐2015) were also similar across study visits, with no difference between WSG vs. WL (Tables 2,3). Thus, there was no GroupWise effect of CNR interventions on dietary quality and the HEI‐2015 scores were similar to those obtained for the U.S. adults [29]. Specifically, expected mean HEI scores range between 53 and 64 depending on population age group vs. the mean (SD) in this study of 56 ± 14. In addition, average fibre intake at V1 was not different between WSG and WL group (17 ± 8 vs. 17 ± 11 g respectively, *p* = 0.4). Furthermore, no significant change in fibre intake was observed in either group at V2 (WSG 19 ± 9 vs. WL 20 ± 7 g, *p* = 0.4).

### 3.5 GM Predictors of Weight Change Response to Nutritional Intervention

**FIGURE 2:**
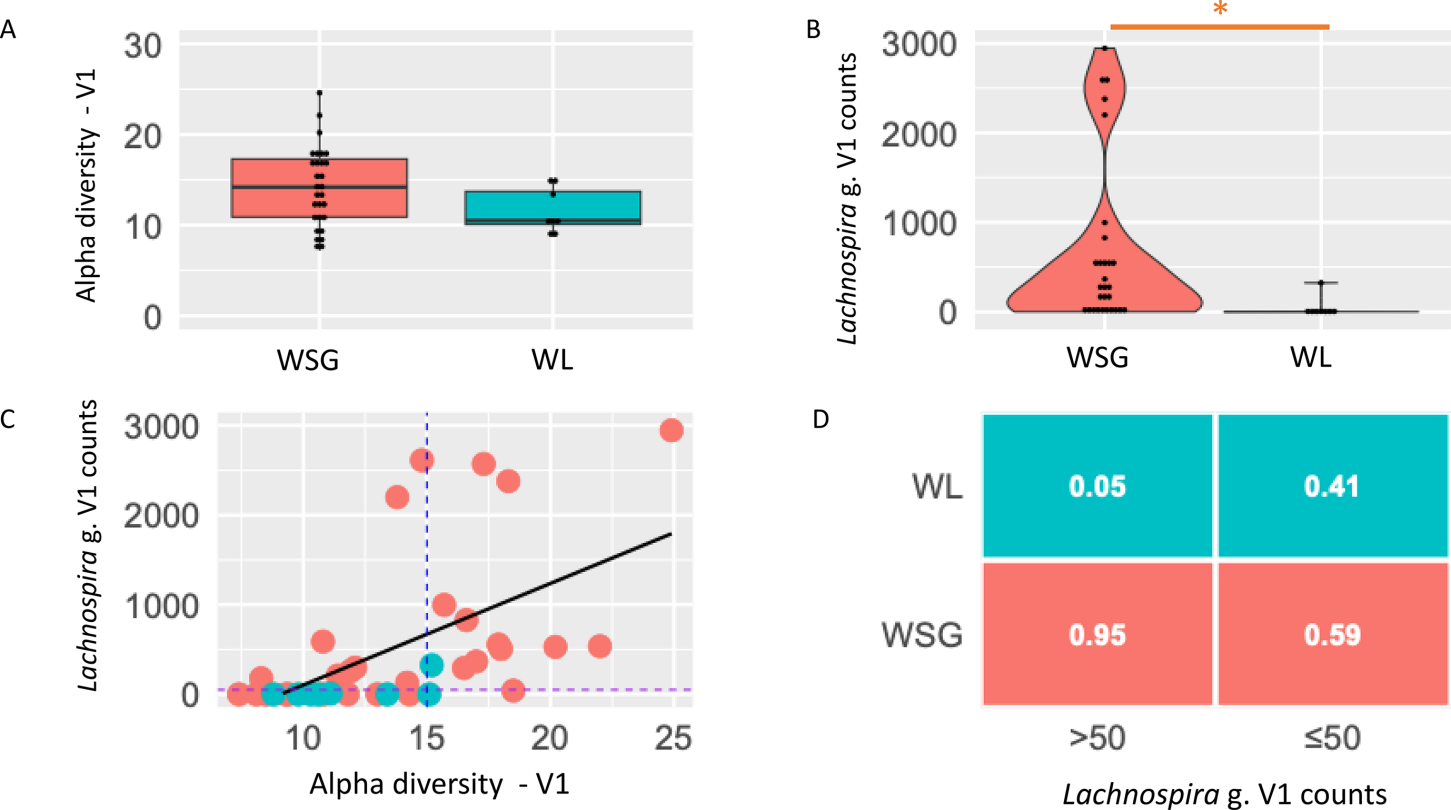
Baseline GM features and weight change response to nutritional intervention. a). Alpha‐diversity (Faith PD) at V1 for participants who maintained/gained weight (WSG, *N* = 29) vs. lost weight (WL, *N* = 8), *p* = 0.14. b). Relative counts for *Lachnospira* genus for WSG (N = 29) and WL (N = 8) at V1 (taxa identified as differentially abundant using ANCOM‐bc, Holm‐Bonferroni adjusted *p*‐value = 0.014). *Lachnospira* genus counts at V1 are higher in WSG vs. WL (log fold change −2.8, 95% CI [−2.0 to −3.6], *p* = 0.01). c). *Lachnospira* genus counts correlate with alpha‐diversity (R_s_ = 0.7, *p* < 0.001). WL (green), WSG (orange). Dashed blue line represents alpha‐diversity Faith PD = 15, Dashed purple line represents *Lachnospira* genus counts at V1 = 50. d). Conditional probability analysis table showing probability of outcome (WSG vs. WL at V2) given *Lachnospira* genus counts at V1. Abbreviations – WSG: Weight stable/gain, WL: Weight loss, V1: visit 1, V2: visit 2.

When comparing alpha‐diversity at V1 between subjects by the weight change category they were assigned at V2 (WSG vs. WL) there was no statistically significant difference at the group level (Figure 2A). No differences in alpha‐diversity at V1 were observed when grouping participants based on diet/weight change groups. Nor were there any associations between alpha‐diversity and other confounding variables including body composition, systemic inflammation, key symptoms, appetite scores, etc. (see Data S1). Similarly, the number of ‘most abundant’ ASVs (those making up 80% of the total detected – see Methods) was also no different at V1 between WSG vs. WL groups (Fisher's Test, ns).

However, closer inspection of the baseline alpha‐diversity distribution reveals that higher values are more common in the WSG (Figure 2A). Using an alpha‐diversity (Faith PD) threshold of 15, a conditional probability analysis reveals that if alpha‐diversity > 15 at baseline, there is an 86% chance of being in WSG vs. only 14% of being in WL group. By contrast, if alpha‐diversity was ≤ 15, there was still 74% chance of being in WSG vs. 26% chance of WL (Data S2).

A total of 552 different ASVs, which mapped to 55 unique genera, were present across WSG and WL patient samples at V1. No ASVs were found to be differentially abundant between the weight change subgroups or any of the other clinical subgroups. By contrast, using taxa analysis, the *Lachnospira* genus was found to be significantly lower at baseline in WL vs. WSG group (log fold change −2.8, 95% confidence interval [−2.0 to −3.6], adjusted *p*‐value: 0.01) (Figure 2B). *Lachnospira* genus (family *Lachnospiraceae*) was also differentially abundant when comparing combined diet/weight change groups i.e., lower in those eating well but still losing weight vs. those eating well and gaining weight (exploratory outcome). No other genera were differentially abundant between weight change subgroups, including *Veillonella* genus (Figure S1). In addition, no genera were found to be differentially abundant between any of the remaining clinical subgroups (sarcopenic status, CRP levels, ALM change).

This result was corroborated when using data from 14 other samples from participants who provided stool samples at V1 only, but then underwent JGH‐CNR interventions and had weight changes recorded. When these data were combined with that from the current study (total 52 participants: 13 WL vs. 38 WSG), *Lachnospira* genus was still significantly lower in WL group: log fold change −2.6, 95% confidence interval [−1.8 to −3.4], adjusted *p*‐value: 0.03.

Using conditional probability analysis, high abundance of *Lachnospira* genus is associated with better weight response e.g., if counts > 50 at V1 the probability of WSG 95% vs. WL 5%. However, if *Lachnospira* counts ≤ 50, it is more difficult to distinguish the two outcomes: probability of WSG 59% vs. WL 41% (Figure 2D). Alpha‐diversity and *Lachnospira* counts do correlate (Figure 2C) but combing both measures does not improve the prediction of weight change over using *Lachnospira* counts alone. If either *Lachnospira* count > 50 OR alpha‐diversity > 15, probability of WSG 91% vs. WL 9%, but if neither condition is present probability of WSG 60% vs. WL 40% (Data S2).

A secondary outcome for this study was to determine predictors of lean mass change. There were no differentially represented ASVs, or genera at V1, between those who went on to lose vs. gain ALM. However, the latter subgroup was based on a small sample size (ALM gain *N* = 8 vs. 4 ALM loss).

### 3.6 Differences in GM Adaptation During Nutritional Intervention

**FIGURE 3:**
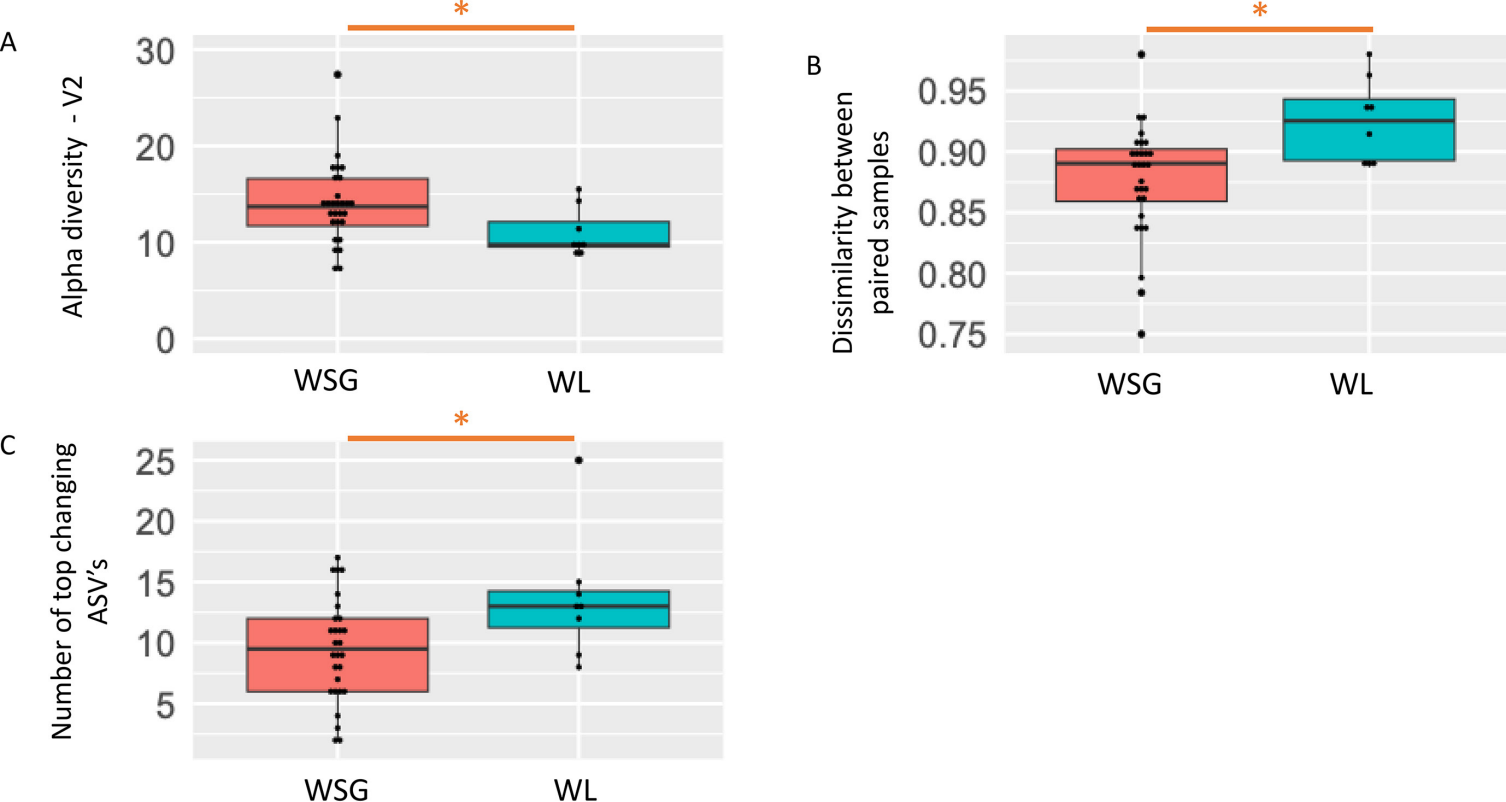
GM differences between weight change groups after nutritional intervention. a). Alpha‐diversity (Faith PD) at V2 is lower in WL (*N* = 8) vs. WSG (*N* = 29) (*p* = 0.05). b). Dissimilarity (beta‐diversity) between paired samples (Jaccard Diversity distance matrix) is lower in WSG (N = 29) vs. WL group (N = 8); *p* = 0.01. c). More top changing sequences in WL vs. WSG group; *p* = 0.05. Abbreviations – WSG: Weight stable/gain, WL: Weight loss, V2: visit 2.

At V2, alpha‐diversity for WL was lower than the WSG group (Figure 3A, Faith PD *p* = 0.05, Observed OTUs *p* = 0.046). There was also greater dissimilarity between WL pairs (V2‐V1) vs. WSG pairs (*p* = 0.01) (Figure 3B). In addition, there was a greater number of ASVs with large changes in abundance (counts) between V1 and V2 between WSG and WL groups (*p* = 0.05) (see Figure 3C). By contrast, there were no differentially represented ASVs, or genera at V2 between WL and WSG groups.

## 4 Discussion

The primary aim of this study was to identify specific features of the gut microbiome from baseline stool samples which could be used to predict the response to individualized dietary intervention in cancer cachexia. The results show that there were no robust differences, at the group level, in baseline diversity between participants who went on to be designated WL vs. WSG. Nevertheless, at the individual level, a higher alpha‐diversity was predictive of positive response to nutritional intervention. A similar pattern was observed with *Lachnospira* genus abundance, but for *Lachnospira* there was also a significant difference in counts at the group level with lower baseline abundance in WL vs. WSG (Figure 2B). Conditional probability analysis confirmed that higher baseline *Lachnospira* abundance was strongly predictive of positive response to nutritional intervention (Figure 2B,D). However, neither low alpha‐diversity nor *Lachnospira* abundance was useful in identifying those who did not respond well to nutritional intervention (WL). Given the complexity of the clinical scenarios in cancer cachexia, it is likely that any single factor (e.g., *Lachnospira* abundance) will have limited predictive capacity if considered in isolation. Thus, larger studies adequately powered to perform multivariate analysis are needed to identify combinations of factors, including *Lachnospira* abundance that can predict poor response to nutritional intervention in cancer cachexia (i.e. WL vs. WSG) more accurately.

The *Lachnospira genus* has been reported to be significantly less abundant in sarcopenic older individuals compared to healthy older controls [30]. *Lachnospira* has also been shown to be significantly reduced in females from the TwinsUK cohort who had reduced appetite scores compared to those who did not [31]. In the current study, this genus was not differentially abundant between subjects with low vs. high appetite scores. However, recognizing that reduced muscle mass and anorexia are two common features in cachexia, and that in the current study, higher *Lachnospira* abundance was associated with better weight gain response to nutritional intervention, further studies are needed to confirm and clarify the predictive capacity of *Lachnospira* genus abundance in cancer cachexia management.

The PANCAX study is the only other longitudinal study in a comparable cancer patient group. It sought to identify baseline GM features that predicted response to jejunal enteral feeding, with oral intake when possible, in cachectic pancreatic cancer patients undergoing palliative chemotherapy. The authors found that higher abundance of the *Veillonella* genus in stool at baseline was associated with subsequent weight stability [18]. Unlike the current study, the PANCAX trial focused exclusively on patients with pancreatic ductal adenocarcinoma. There were also important differences in the bioinformatics pipeline used for differential abundance testing and the ANCOM‐bc methodology used in the current study which is now preferred for GM analysis [25]. There is evidence that there may be cancer‐specific GM profiles, for instance, in patients who develop colorectal cancer [32]. Thus, it is certainly possible that there are cancer type or cancer treatment‐specific patterns of GM adaptation to nutritional intervention (especially enteral feeding) which were not evident in the current study. However, there was no difference in abundance of *Veillonella* genus at V1 between WL and WSG in the current study (Figure S1).

The secondary aim of the study was to identify potentially important differences in the GM that emerged (at V2) after nutritional intervention in both WSG and WL groups. The WL group demonstrated lower alpha‐diversity vs. WSG (Figure 3A) and there were greater global changes in the microbial population in WL (Figure 3B,C) at V2. The appearance of a less diverse and less stable GM in subjects who lost weight (WL), developed over the 6 weeks of CNR‐JGH intervention, despite similar improvements in overall dietary metrics (protein and energy intake) and no differences in dietary quality (Table 3). Reduced diversity has been shown to be associated with disease states in many clinical conditions [33], but it is not clear from the results of this study whether these differences in GM diversity and population stability at V2 were driven by some aspect of the CNR‐JGH intervention or the result of other disease‐related factors. Further studies are needed to confirm whether these features are a consistent finding in the WL group and if so, whether therapies to increase GM diversity may be of benefit to improve response to nutritional intervention in cancer cachexia.

The current study has several strengths and describes a cohort of 38 well‐characterised patients attending a specialised cachexia clinic (CNR‐JGH) delivering individualized interventions, including dietetic support from experienced oncology dietitians. The participants were followed closely to measure changes in dietary intake and GM composition and a range of other relevant clinical features. Rigorous methodology for microbiome sequence analysis and statistical testing was employed including use of technical controls for quality assurance and stringent bioinformatic analyses methods. The majority of participants in the current study had established cancer cachexia by standard criteria [26] at baseline and most were in the two worst prognostic categories using the cancer weight loss grading system [27] (Table 1).

However, several limitations to this study should be recognized. Whilst the study was conceived to identify major patterns of GM that predict response to nutritional intervention irrespective of cancer type, the wide range of different cancer types included may have been a disadvantage, especially given the final size of the overall study group. As mentioned above, there is at least some evidence for cancer‐specific patterns of change in GM [32] and thus it is possible that important cancer‐specific differences in responses to nutritional intervention may have been obscured. Moreover, the study cohort was not large enough to perform subgroup analyses required to clarify some of these questions. Other confounding factors include, the range of different cancer treatments received by the participants during the study period which may impact the GM and the variation in baseline diet and other medication use. Participants included were at different stages of their cancer treatment and some participants had already received some form of dietary counselling before entering the study. Despite these caveats, the results suggest that abundance of *Lachnospira* genus may potentially be a predictor of weight change response to nutritional intervention in cachexia and deserves further study.

## 5 Conclusion and Future Directions

More robust predictors of response to dietary intervention and a wider range of individualized approaches to enhance weight gain with dietary interventions would be of major therapeutic benefit in the overall management of cancer cachexia. The current study was designed to identify potential GM‐related biomarkers of response to individualized nutritional interventions in individuals with cancer cachexia being treated by an expert multidisciplinary team. The results suggest GM analysis may help predict patients' responses to nutritional intervention. Confirmatory studies are needed to determine if *Lachnospira* genus abundance is a robust predictive biomarker of positive weight change response to dietary intervention. The early identification of individuals who will not respond well to nutritional intervention is a priority in cancer cachexia management and future studies using other clinical and biochemical features in multivariate modelling may be needed to enhance the predictive capacity of *Lachnospira* abundance alone. Given the observation that the WL group GM was less diverse and more unstable after CNR‐JGH intervention, additional targeted dietary interventions (e.g. including use of probiotics) to promote greater diversity and GM stability may also prove beneficial.

## Author Contributions

R.T.J. and R.N. conceived study. K.D. helped guide the 16S rRNA sequencing methodology and interpretation and S.C. provided valuable feedback on analysis and reporting of the nutritional component of the study. R.N. and C.V.B. performed all dietary counselling, collected, and processed diet records. M.K. assembled all additional data. R.T.J. and R.N. wrote the manuscript and R.N. performed statistical analyses. All authors edited the final draft of the manuscript.

## Ethics Statement

This study has been approved by the appropriate ethics committee and has therefore been performed in accordance with the ethical standards laid down in the 1964 Declaration of Helsinki and its later amendments. All patients gave their informed consent prior to their inclusion in the study.

## Conflicts of Interest

The authors declare no conflicts of interest.

## Supporting information


**Data S1** Supporting Information.

**Data S2** Supporting Information.

**Figure S1** Relative counts for selected taxa between weight change groups at V1. Relative counts for *Veillonella* genus for WSG (*N* = 29) and WL (*N* = 8) at V1. Dots represent participants. Abbreviations – WSG: Weight stable/gain, WL: Weight loss, V1: visit 1, V2: visit 2.
**Figure S2** Heatmap of relative counts for all genera assigned between weight change groups. A heatmap of relative counts at V1 for all genera assigned for weight change group. Green dotted line divides WSG (*N* = 29) vs. WL (W = 8). Purple arrow indicates *Lachnospira* genus. Green arrow indicates *Veillonella* genus. Abbreviations – WSG: Weight stable/gain, WL: Weight loss.
**Figure S3** Per individual changes in alpha‐diversity across visits between weight change groups. Per individual changes in alpha‐diversity (Faith PD) across visits (V1 and V2). WSG (*N* = 29) in orange, WL (*N* = 8) in teal. No difference in mean change between groups (WSG − 0.07 vs. WSG ‐0.6, *p* = 0.80). Abbreviations – WSG: Weight stable/gain, WL: Weight loss, V1: Visit 1.

**Table S1** Definitions of clinical categories.
