# Supplementary material for: Stool Microbiome Features and Weight Change Response to Treatment for cancer cachexia
Source: J Cachexia Sarcopenia Muscle. 2025 May 5;16(3):e13816. doi: 10.1002/jcsm.13816 (PMC12052804; doi:10.1002/jcsm.13816)
Supplement: Supplementary file 1 — Data S1 Supporting Information. [file JCSM-16-e13816-s004.docx]

**Supplementary Material - Detailed Methods**

**Body composition measurements (DXA)**

Dual energy x-ray absorptiometry (DXA) scans were used to assess total body and regional measurements of the arms and legs. Appendicular lean mass (ALM), a proxy for skeletal muscle mass, was calculated as the sum of arms and legs’ lean soft tissue mass (without bone). ALM, total fat mass, and total lean mass were divided by height^2^ to derive their respective indices: ALM index (ALMi), fat mass index, and lean mass index. To control for differences in sex, age, and ethnicity, z-scores for ALMi, fat mass index, and lean mass index was calculated by using reference data from the National Health and Nutrition Examination Survey (NHANES) 1999-2004 ^(1)^ (based on the United States population given there are no Canadian references at the time of this manuscript). Change in arms (lean/fat), legs, total lean, and total fat between visits were calculated. Change in appendicular lean mass between visits (total and rate as measured by total/100 days) were also calculated. The above measurements were used when testing associations between alpha diversity and continuous metadata.

Participants were categorized based on sarcopenic status using ALMi cut-off points defined by recent sarcopenia definition from a Canadian cohort^(2)^. Participants were also categorized based on whether they gained appendicular lean mass and total lean body mass (see Supplementary Table for definitions).

Only DXA scans conducted within 30 days of a clinic visit were included for analysis.

**DNA extraction and 16S rRNA sequencing**

DNA extraction from stool samples was performed at the Microbiology department at the Jewish General Hospital. DNA extraction was performed upon receipt of sample using EazyMag (Biomerieux, Saint-Laurent, Quebec, Canada), following stool isolation protocol (100uL input, 100uL output), as used previously at the same site^(3)^. In brief, samples were vortexed and centrifuged to remove unwanted stool constituents (i.e., fiber content, undigested food materials). The clear supernatant which includes bacterial DNA was extracted through EasyMag, a semi-automated robot that performs nucleic acid extraction (through binding the nucleic acids to magnetic silica particles). Nucleic acid concentration was quantified with the spectrophotometer Nanodrop and the purity of nucleic acids was assessed by calculating the ratio of absorbance at different wavelength (A_260/280_). The 100uL DNA extracts were frozen at -70C and later sent on dry ice to Genome Quebec for 16S rRNA sequencing. PCR amplification of the 4^th^ hypervariable region (V4) of the 16S ribosomal RNA (rRNA) was performed using the 515/806R primer set. 16S rRNA sequencing was performed on Illumina MiSeq PE250 using golay barcodes, producing 250bp paired-end reads.

**QIIME2 16S rRNA gene sequence data processing**

First, sequences were demultiplexed. The quality score was high at each base pair (bp) position with a median score of 35 at bp 250. Thus, sequences were not trimmed further. Quality filtering was performed using DADA2, a pipeline for detecting and correcting Illumina amplicon sequence data^(4)^. Around 77% of the sequences were retained after passing the DADA2 command.

DADA2 steps include the filter and trim step: filtering out PhiX reads (control libraries generated from the PhiX virus), trimming all reads to 250 bp, filtering out reads that with at least one ambiguous nucleotide, and discarding reads with expected errors and singleton reads. Although PhiX reads were already filtered out by the sequencing facility, this additional step serves as extra quality control to filter out any missed PhiX reads. Next, DADA2 dereplicates reads (groups all reads with the same sequence together to reduce computation time during downstream analysis). Sample composition is then inferred by learning error rates using an algorithm, to make a sequence table (counts of sequences per each sample). The quality control process also filters any chimeric sequences (hybrid products between multiple parent sequences) that can be falsely interpreted as novel organisms (as this can inflate apparent diversity).

A rooted phylogenetic tree was constructed to calculate certain diversity measures that account for phylogenetic relationships of sequences (for example, Faith Phylogenetic Diversity requires a phylogenetic tree). To create this tree, sequences were first aligned to infer a phylogenetic tree using the ‘mafft’ method (multiple sequence alignment program in QIIME). FastTree command in QIIME was applied to infer a phylogenetic tree from the multiple sequence alignment generated by the previous step.

Reads per sample ranged from ~18,500 to ~67,500. A sampling depth of ~18,500 reads per sample was applied as a pre-step for the diversity analyses to control for falsely affecting diversity measures.

**Diversity Analyses**

Alpha diversity analyses were used to investigate stool microbiome diversity between different clinical groups (i.e. WSG vs WL) at each clinic visit (V1 and V2) using 4 common indices included in the core package of QIIME 2: Shannon, Number of distinct features (Observed OTU’s), **Faith’s phylogenetic diversity (**Faith PD), and Pielou’s Evenness indices. ‘Alpha-group-significance’ QIIME 2 command was used to test associations between discrete metadata categories and alpha diversity data, while the ‘alpha-correlation’ command was used for continuous metadata. ‘Pairwise-differences’ command was used to determine whether alpha diversity changed significantly between paired stool samples from the same individual between Visit 2 and Visit 1 (e.g. pre- and post- CNR-JGH interventions) across different clinical categories.

Beta-diversity (B-diversity) was measured through Jaccard distance, Bray-curtis, Unweighted Unifrac, and Weighted Unifrac indices. Beta diversity indices calculate a distance between *a pair* of samples (rather than calculating a value for each sample as in alpha-diversity metrics), with larger values indicating more dissimilar pairs. ‘Pairwise-distances’ command in QIIME2 operates on this distance matrix to assess the distance between paired samples to test whether these paired differences are significantly different between different categories (i.e. whether paired samples V1-V2 were different between WSG vs WL).

In addition to significance testing for each diversity metric, a concordance threshold was set requiring a minimum of two out of the four diversity metrics to be statistically significant for an overall difference in diversity to be declared.

**Quality Control and stool sample inclusion**

1. *Testing DNA extraction reproducibility:*

One stool sample from a healthy volunteer was homogenized, aliquoted into vials and frozen at -80^0^C. This sample was processed without the use of Omnigene. DNA extraction from each aliquot was performed whenever an incoming stool sample from a participant was processed. From December 2016 to June 2018, 64 aliquots were extracted and later sequenced. Alpha diversity and assigned taxonomy at the genus level were consistent across these samples, except for two aliquots which showed differing relative abundance of taxa (qualitative assessment).

1. *Testing PCR sequencing reproducibility (within-run):*

Two aliquots of DNA extracts from the same 5 subjects were sequenced twice on the same run. Each pair of samples from an individual exhibited similar alpha diversity as well as assigned taxonomy at the genus level, showing greater similarity between pairs from the same individual than between different individuals.

1. *Comparison of taxonomy results obtained with two different sequencers to track drift between sequencing runs:*

A subset of samples (N=39) was sequenced at the Knight Lab facility at the University of California San Diego (UCSD), with sequences trimmed at 150 bp, differing from the 250 bp trimming at Genome Quebec. Despite these methodological differences, the assigned taxonomy was very similar, with comparable proportions of assigned taxa in each sample between the two datasets (i.e. Genome Quebec and UCSD).

1. *Testing background contamination false positive reads using negative controls:*

Five negative controls were sequenced with the rest of the participant samples. Negative controls consisted of the PCR mixture including buffer, DSMO, nucleotides, enzymes, primers and water but without a DNA template. These controls showed a total of five different sequences with a median of ~30 reads per sequence, ranging from 13-600 reads per sample, consistent with expected results from the Microbiome Quality Control Project.

**References**

1. Kelly TL, Wilson KE, Heymsfield SB (2009) Dual energy X-Ray absorptiometry body composition reference values from NHANES. *PloS one* **4**, e7038.

2. Tessier AJ, Wing SS, Rahme E *et al.* (2019) Physical function‐derived cut‐points for the diagnosis of sarcopenia and dynapenia from the Canadian longitudinal study on aging. *Journal of Cachexia, Sarcopenia and Muscle* **10**, 985-999.

3. Brukner I, Longtin Y, Oughton M *et al.* (2015) Assay for estimating total bacterial load: relative qPCR normalisation of bacterial load with associated clinical implications. *Diagnostic Microbiology and Infectious Disease* **83**, 1-6.

4. Callahan BJ, McMurdie PJ, Rosen MJ *et al.* (2016) DADA2: High-resolution sample inference from Illumina amplicon data. *Nature methods* **13**, 581-583.
