# Supplementary material for: Stool Microbiome Features and Weight Change Response to Treatment for cancer cachexia
Source: J Cachexia Sarcopenia Muscle. 2025 May 5;16(3):e13816. doi: 10.1002/jcsm.13816 (PMC12052804; doi:10.1002/jcsm.13816)
Supplement: Supplementary file 3 — Figure S1 Relative counts for selected taxa between weight change groups at V1. Relative counts for Veillonella genus for WSG (N = 29) and WL (N = 8) at V1. Dots represent participants. Abbreviations – WSG: Weight stable/gain, WL: Weight loss, V1: visit 1, V2: visit 2. Figure S2 Heatmap of relative counts for all genera assigned between weight change groups. A heatmap of relative counts at V1 for all genera assigned for weight change group. Green dotted line divides WSG (N = 29) vs. WL (W = 8). Purple arrow indicates Lachnospira genus. Green arrow indicates Veillonella genus. Abbreviations – WSG: Weight stable/gain, WL: Weight loss. Figure S3 Per individual changes in alpha‐diversity across visits between weight change groups. Per individual changes in alpha‐diversity (Faith PD) across visits (V1 and V2). WSG (N = 29) in orange, WL (N = 8) in teal. No difference in mean change between groups (WSG − 0.07 vs. WSG ‐0.6, p = 0.80). Abbreviations – WSG: Weight stable/gain, WL: Weight loss, V1: Visit 1. [file JCSM-16-e13816-s002.pptx]

## Slide 1
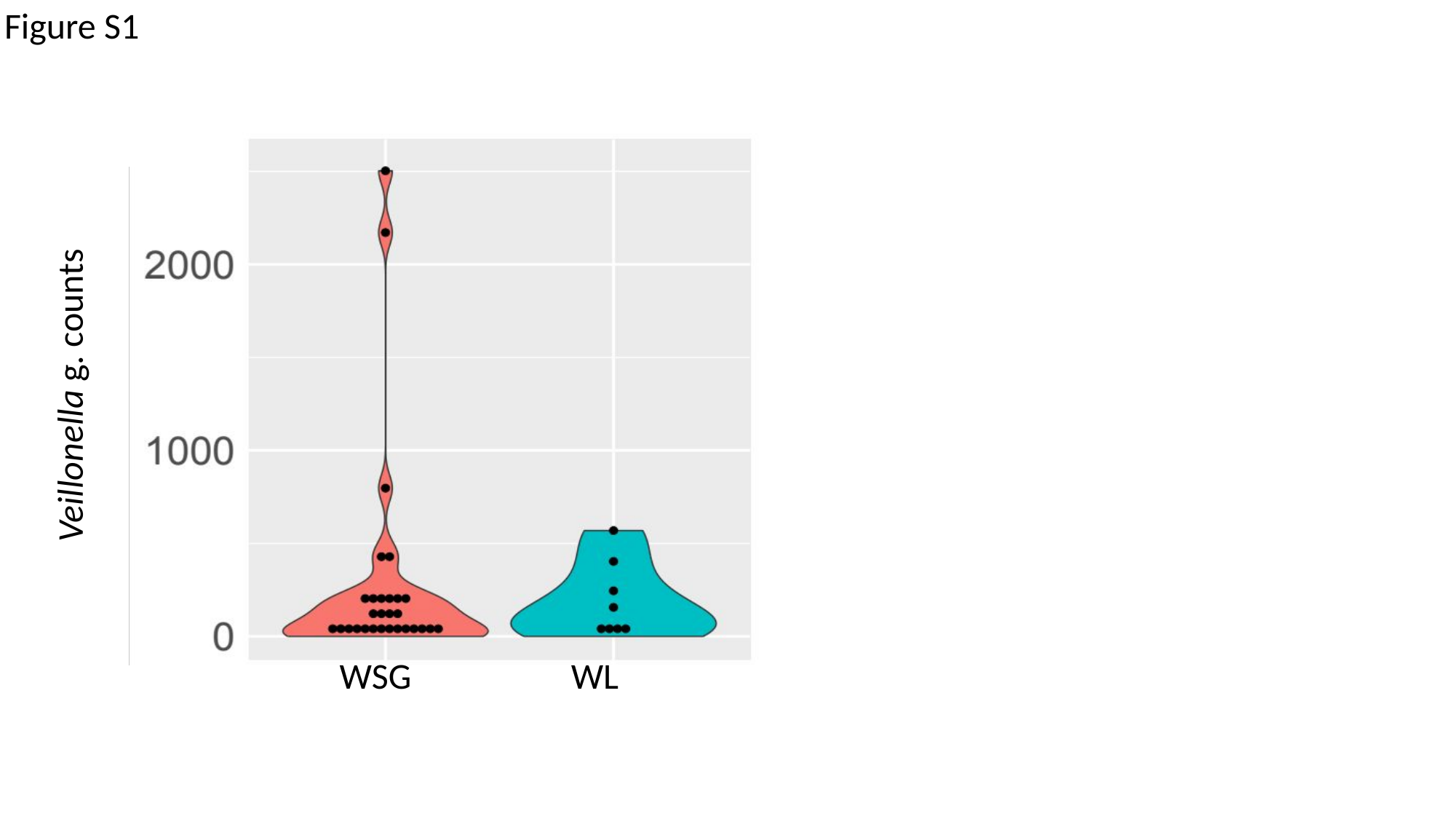

Figure S1
Veillonella g. counts
WSG
WL

## Slide 2
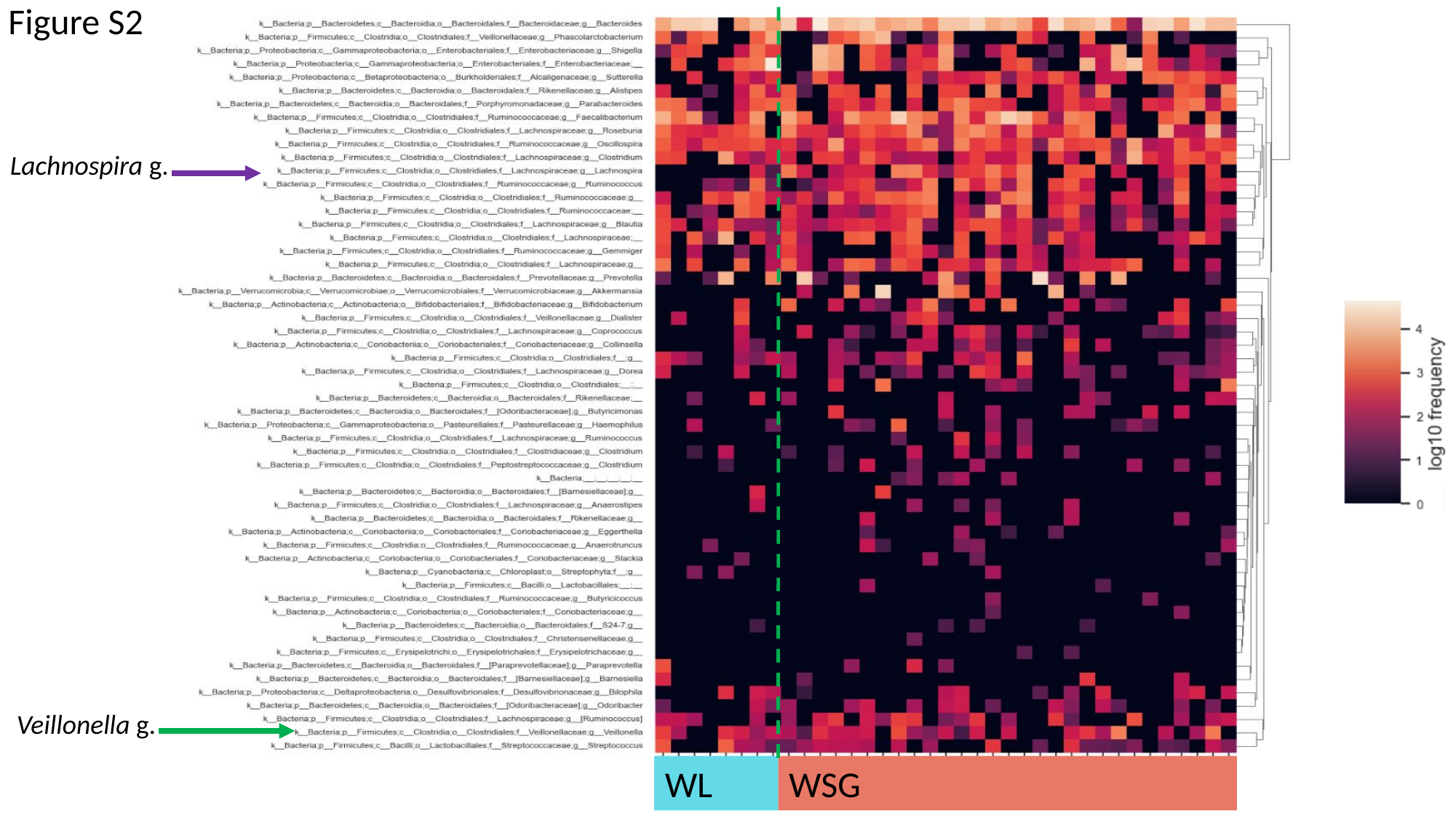

Figure S2
WL
WSG
Lachnospira g.
Veillonella g.

## Slide 3
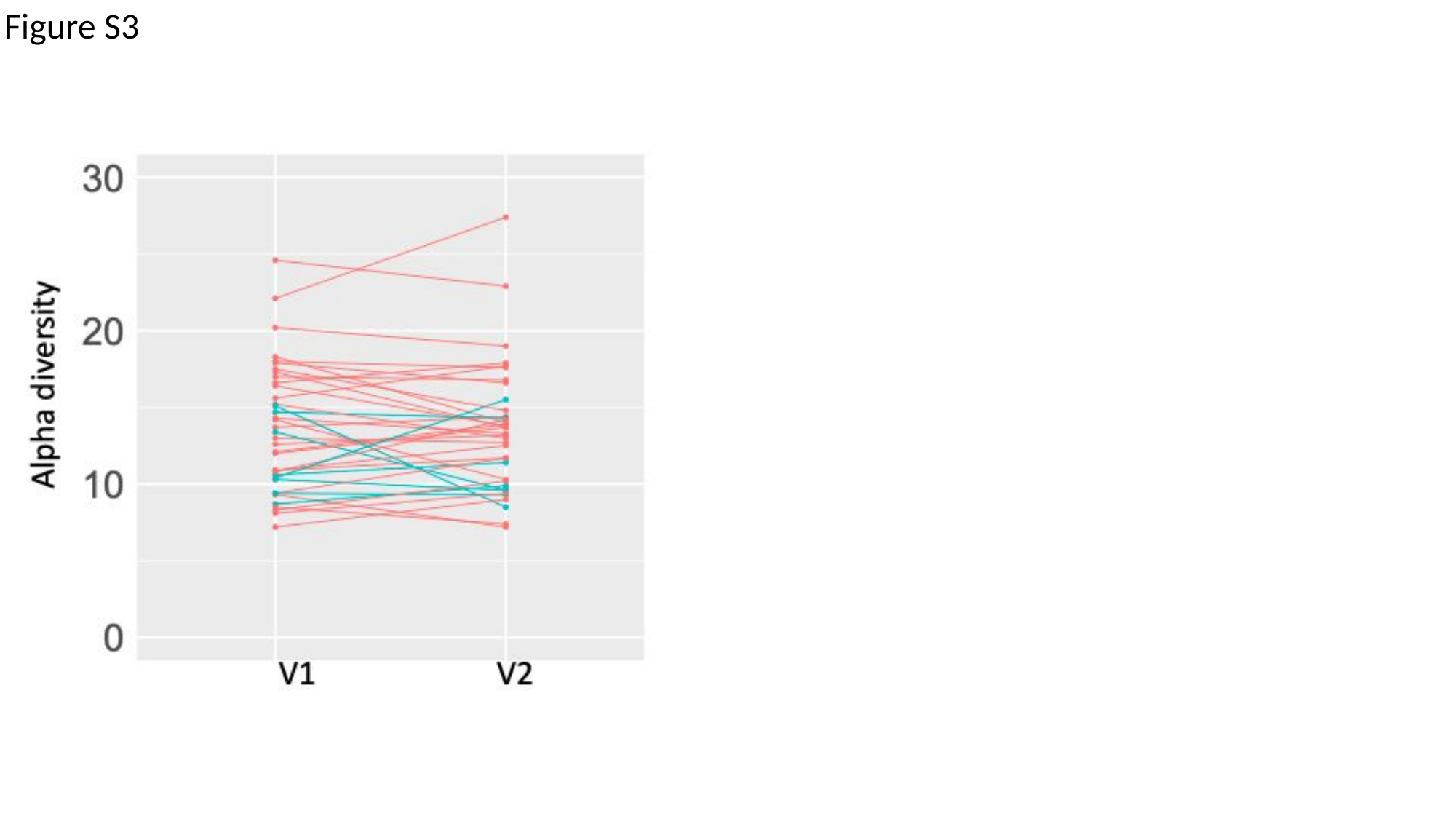

Figure S3
