# Supplementary material for: Stool Microbiome Features and Weight Change Response to Treatment for cancer cachexia
Source: J Cachexia Sarcopenia Muscle. 2025 May 5;16(3):e13816. doi: 10.1002/jcsm.13816 (PMC12052804; doi:10.1002/jcsm.13816)
Supplement: Supplementary file 4 — Table S1 Definitions of clinical categories. [file JCSM-16-e13816-s001.docx]

Table S1: Definitions of clinical categories

| **Clinical Group** | **Categories** | **Definition** |
| --- | --- | --- |
| **BMI** | Low | <22 kg/m^2^ |
|  | Medium | 22-28 kg/m^2^ |
|  | High | >=29 kg/m^2^ |
| **Weight change** | WL | >1 kg weight loss at V2 |
|  | WSG | ≤1 kg weight loss at V2 |
| **Weight/Diet** | Diet Responders | Eating well, and gained/maintained weight at V2 |
|  | Diet Resistant | Eating well, but lost weight at V2 |
| **Weight change pre-6 week to V1** |  | Reported weight change from ~6-week pre-V1 to V1 |
| **Weight loss trajectory** |  | Losing at 6 months and at 6 weeks pre-V1 |
| **Energy intake** | Low | < 25 kcal/kg |
|  | Moderate | 25-30 kcal/kg |
|  | High | >30 kcal/kg |
| **Protein intake** | Low | 1.3 g/kg + |
|  | Moderate | 1-1.3 g/kg |
|  | High | < 1 g/kg |
| **Appendicular lean mass change** | Gain | > 0.5 kg increase |
|  | Non-gain |  |
| **Total lean body mass change** | Gain | > 0.7 kg increase |
|  | Non-gain |  |
| **CRP categories** | High | > 10 mg/dL (i.e. log CRP >1) |
|  | Low | < 10 mg/dL |
| **Symptom score** | *Score out of 10, for anorexia, pain, weakness, nausea, vomiting, diarrhea, constipation, fatigue, shortness of breath, depression* | |
|  | Low | 0-3 |
|  | Moderate | 5 to 6 |
|  | High | 7 to 10 |
| **Other symptoms** | presence of (*yes/no responses)*: | |
|  | dry mouth, mouth sores, problems swallowing, smell disturbances, taste issues, early satiety | |
| **Medication use** | metformin, other diabetic medication, proton pump inhibitors, statins, laxatives, and antibiotics, loperamide (anti-diarrheal medication) | |

Abbreviations – BMI: body mass index, WSG: Weight stable/gain, WL: Weight loss, ALM: appendicular lean mass, V2: Visit 2, CRP: C-reactive protein

**References for Tables**

1. Fearon K, Strasser F, Anker SD *et al.* (2011) Definition and classification of cancer cachexia: an international consensus. *The lancet oncology* **12**, 489-495.

2. Martin L, Senesse P, Gioulbasanis I *et al.* (2015) Diagnostic criteria for the classification of cancer-associated weight loss. *Journal of clinical oncology* **33**, 90-99.

3. Douglas E, McMillan DC (2014) Towards a simple objective framework for the investigation and treatment of cancer cachexia: the Glasgow Prognostic Score. *Cancer treatment reviews* **40**, 685-691.

4. Tessier AJ, Wing SS, Rahme E *et al.* (2019) Physical function‐derived cut‐points for the diagnosis of sarcopenia and dynapenia from the Canadian longitudinal study on aging. *Journal of Cachexia, Sarcopenia and Muscle* **10**, 985-999.
